# Experiences of practicing surgical neuro-oncology during the COVID-19 pandemic

**DOI:** 10.1007/s11060-020-03489-6

**Published:** 2020-04-10

**Authors:** Yuan-Jun Hu, Jian-min Zhang, Zhong-ping Chen

**Affiliations:** 1 Department of Neurosurgery/Neuro-Oncology, State Key Laboratory of Oncology in South China, Collaborative Innovation Center for Cancer Medicine, Sun Yat-Sen University Cancer Center, Guangdong, 510060 People’s Republic of China; 2 Department of Neurosurgery, Second Affiliated Hospital of Zhejiang University College of Medicine, Hangzhou, China

To the Editor,

Since the beginning of 2020, the novel coronavirus disease known as COVID-19 emerged to become an unprecedented global pandemic. By April 5, 2020, the confirmed cases have exceeded 1 million and 57, 206 deaths have been reported globally. The latest study from China indicated that cancer patients were at higher risk of COVID-19 infection and had poorer outcomes than the general population [1]. Patients with tumors of the central nervous system face multiple challenges, not just due to their immunocompromised state, which makes them more susceptible to infection, but also due to the need for extreme caution when performing treatments including surgery. We are also faced with significantly altered hospital systems, as well as shortage of medical supplies. Here, we share our current experience in the surgical management of patients with CNS tumors during the COVID-19 pandemic.

Our first strategy is triage. Patients with CNS tumor were classified at initial visit according to their diagnoses and clinical status. Patients who required routine follow-up were encouraged to take advantage of virtual healthcare options using online medical platforms. For the patients with tumors that are more likely benign or low grade gliomas, and if they are asymptomatic, elective surgery would be postponed until a safer time. Meanwhile, for patients with malignant tumors such as higher grade gliomas or benign tumor with severe symptoms, surgical intervention should be scheduled in a timely manner, since delay in surgery could reduce the chance of treatment success. For emergency cases, such as patients with acute hydrocephalus or cerebral herniation, surgery must be arranged immediately. In these cases, the results of COVID-19 testing may not be available prior to surgery, and thus the surgery should be performed under strict precautions to minimize possible exposure to the novel coronavirus.

Hierarchical protection is another important tactic in our current practice. We now adopt three levels of protection to avoid occupational exposure to novel coronavirus during surgery. Based on current Chinese guidelines for aerosol transmissible diseases, precautions for health care professionals are currently categorized into 3 levels, with level 1 being the lowest level of precaution, and level 3 being the highest. Level 1 precautions requires the use of a surgical cap, surgical face mask, protective gown and gloves. Level 3 precaution requires surgical cap, N95 face mask, goggles, face shield, full face piece respirator, protective gown, gloves. All patients admitted for surgery must undergo “COVID-19 screening”, including contact tracing, symptoms interrogation, novel coronavirus nucleic acid and antibody test, and chest CT scan. Specific precautions will then depend on the results of pre-procedural screening. A variety of personal protective equipment (PPE) are advised to choose for each level [2]. To be specific, patients who are confirmed COVID-19 negative could be operated under level 1 precautions as in low risk areas. For patients who are suspected of having COVID-19 but who require emergency surgery, or patients from a high risk area, the tertiary health care facility must be equipped to perform the surgery. Patients who are COVID-19 positive should be transferred to the designated hospitals for accepting COVID-19 positive patients, with the exception of emergency cases, in which case the operation should be carried out under level 3 precautions [3]. Besides appropriate PPE, a negative pressure operating room and corresponding intensive care unit (ICU) support are indispensable in the management of these patients.

**Fig. 1:**
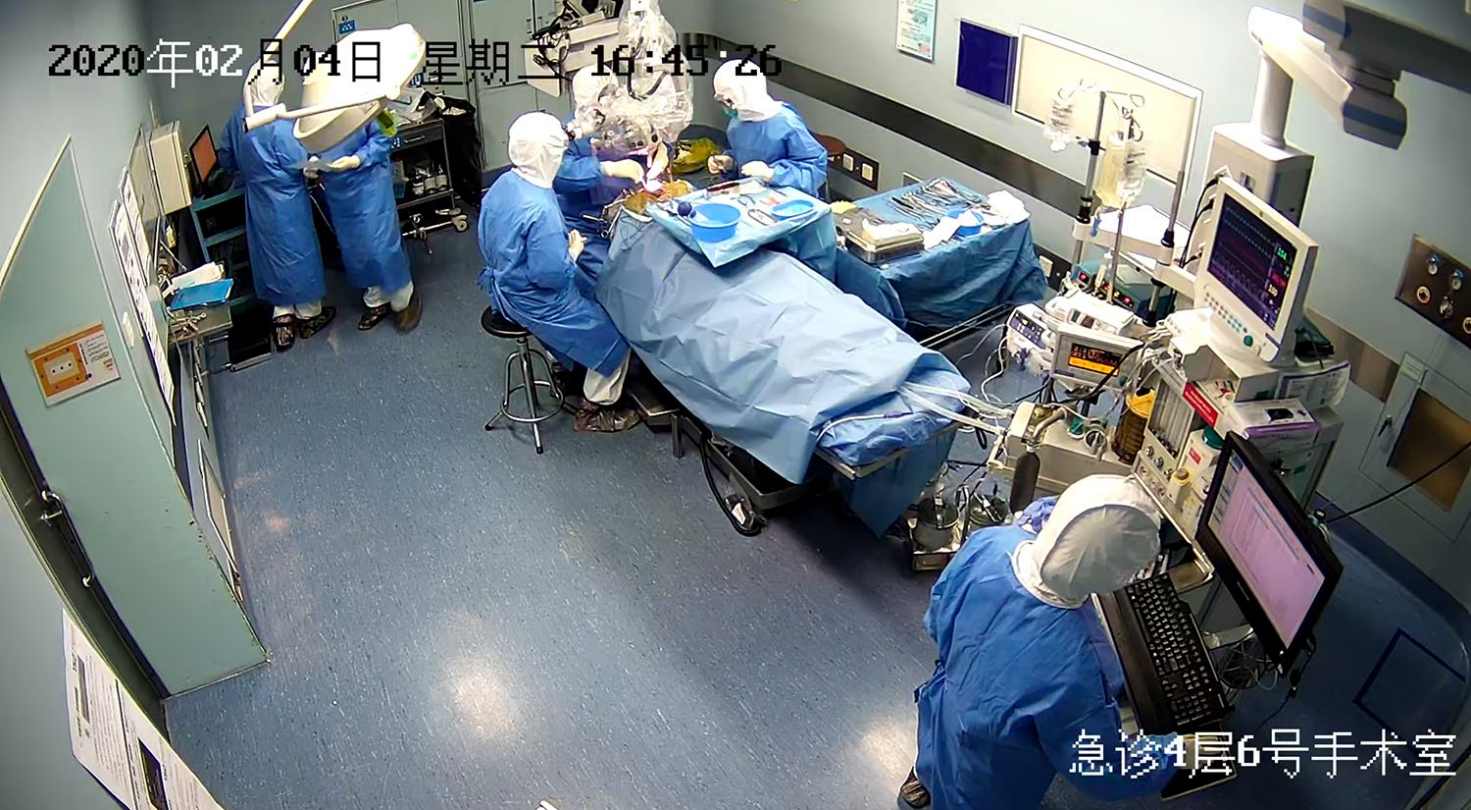
Operation for tumor of the third ventricle in a patient who was suspected of having COVID-19 infection

We now describe a case with suspected COVID-19 infection who received surgery at the Second Affiliated Hospital of Zhejiang University College of Medicine. An 8-year-old boy from Wenzhou, a COVID-19 high risk region, presented to the emergency with a 3-day history of headaches, dizziness, intermittent nausea, and vomiting. Brain MR showed a tumor that was entirely confined within the third ventricle, causing obstructive hydrocephalus. The patient’s COVID-19 screening was negative. Due to progressive symptoms of intracranial hypertension, craniotomy for tumor resection was scheduled for the day after admission. The child then developed symptoms of cough, sneezing, and a mild rise in temperature to 37.6 °C. The patient was strongly suspected of having COVID-19 even though chest CT scan was negative and the initial nucleic acid test demonstrated a negative result. The surgery was thus performed under the level three precautions in a negative pressure operating room (Fig. 1). The child has an uncomplicated recovery after surgery, and COVID-19 was eventually ruled out after a 14-day quarantine.

Finally, we have some additional tips for conducting surgery under level 3 precautions. A comfortable goggle and use of antifogging agent are very helpful. In addition, surgeons should avoid unnecessary conversation and perform the surgery as gently as possible to prevent splashing blood in a negative pressure operating room.

Neuro-oncology patients may become infected with COVID-19, and thus surgery for these patients cannot be avoided. As neurosurgeons, it is crucial that we do our utmost to care for our patients while ensuring our own safety at the same time. In these challenging times, we have faith that through our concerted efforts, this pandemic will be overcome soon.
